# Microchips in Medicine: Current and Future Applications

**DOI:** 10.1155/2016/1743472

**Published:** 2016-06-07

**Authors:** Adam E. M. Eltorai, Henry Fox, Emily McGurrin, Stephanie Guang

**Affiliations:** Brown University, Providence, RI 02903, USA

## Abstract

With the objective of improving efficacy and morbidity, device manufacturers incorporate chemicals or drugs into medical implants. Using multiple reservoirs of discrete drug doses, microchips represent a new technology capable of on-demand release of various drugs over long periods of time. Herein, we review drug delivery systems, how microchips work, recent investigations, and future applications in various fields of medicine.

## 1. Introduction of Drug Delivery Systems

As drug therapies become increasingly complex and effective in treating disease, the development of delivery systems has overcome challenges of achieving stable release rates, drug concentrations, and being at a specific site of action [1]. Traditional routes of administration, such as oral capsules or intravenous infusion, encounter problems in maintaining drug concentrations within the therapeutic window, wherein the drug is above a threshold for efficacy but not toxic to the patient. Thus, the design of delivery systems initially focused on attaining a sustained release of drug over a time interval. Much of this work focused on polymers and their material properties that allow for steady-state diffusion of drug out of the polymer or degradation of the polymer itself over time [2, 3]. In addition to sustained release, pulsatile delivery at variable time intervals is necessary for compounds, such as insulin or hormones of the anterior pituitary, for physiological functions that follow either circadian rhythm or a time structure [4, 5].

With advancement in technology, implantable controlled-release systems for drug delivery have emerged as a promising new class of drug formulation to translate pharmacological effect into clinical practice. Implantable drug delivery systems (IDDS) are currently grouped into three classifications: biodegradable/nonbiodegradable implants, implantable pump systems, and the newest atypical class of implants [6]. Biodegradable and nonbiodegradable implants are available as reservoir and matrix systems, which exhibit release kinetics based on system and surrounding parameters [7]. However, these formulations are not suitable for drugs that are unstable* in vivo* and need to be hermetically sealed, especially since new protein-based drugs become unstable upon water penetration. Some controlled-release microfluidic pumps, valves, and channels have been developed that utilize moving parts such as a pneumatic piston or electroosmotic pumping [8, 9]. However, limitations of drug instability, complexity of fabrication, and breakdown of moving parts hinder clinical translation of microfluidics [10].

As a result, the field of microfabrication demands the need for a new class of controlled-release delivery system of intelligent, programmable microelectronics. Microchips are capable of complex release patterns, simultaneously constant and pulsatile, increased accuracy, and isolation of the drug from the outside environment [11].

## 2. Overview of Microchips

### 2.1. Design and Components

**Figure 1:**
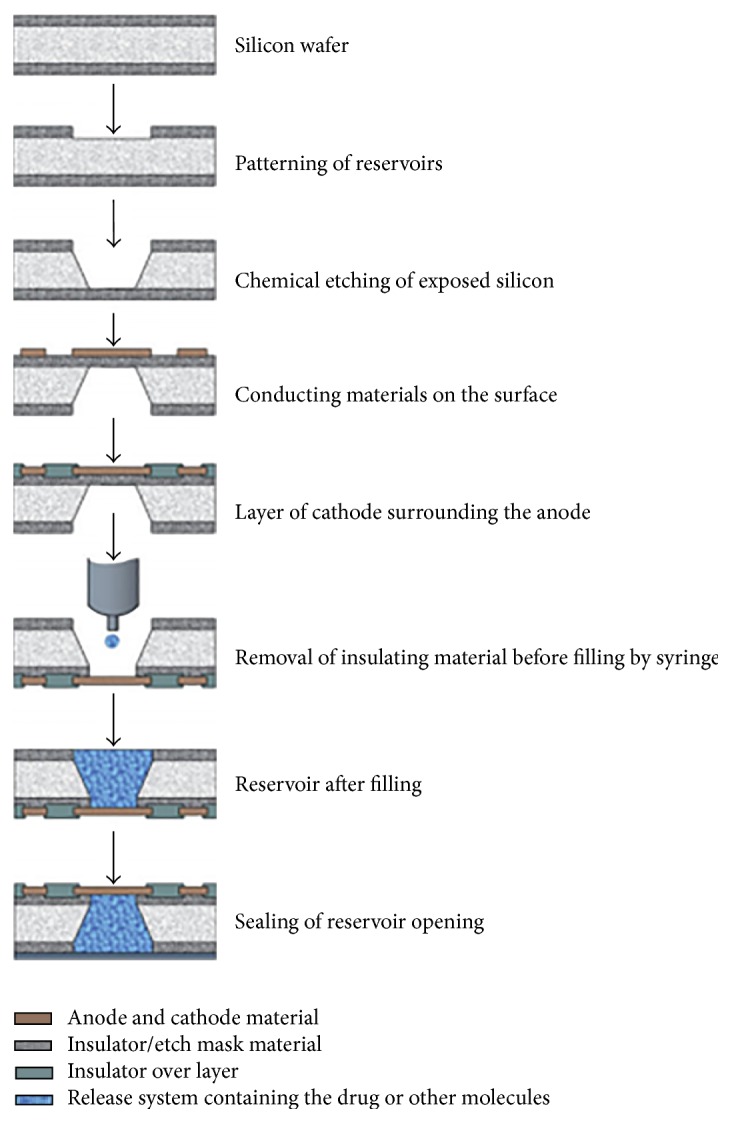
Microchip fabrication steps [23].

**Figure 2 fig2:**
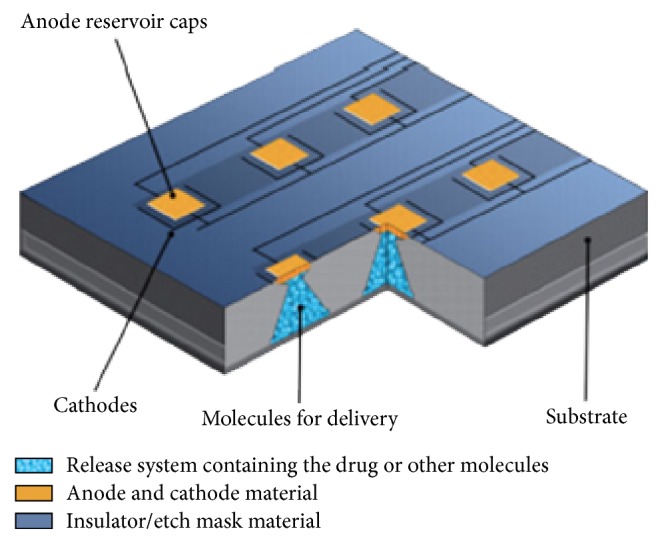
Microchip device schematic [23].

**Figure 3:**
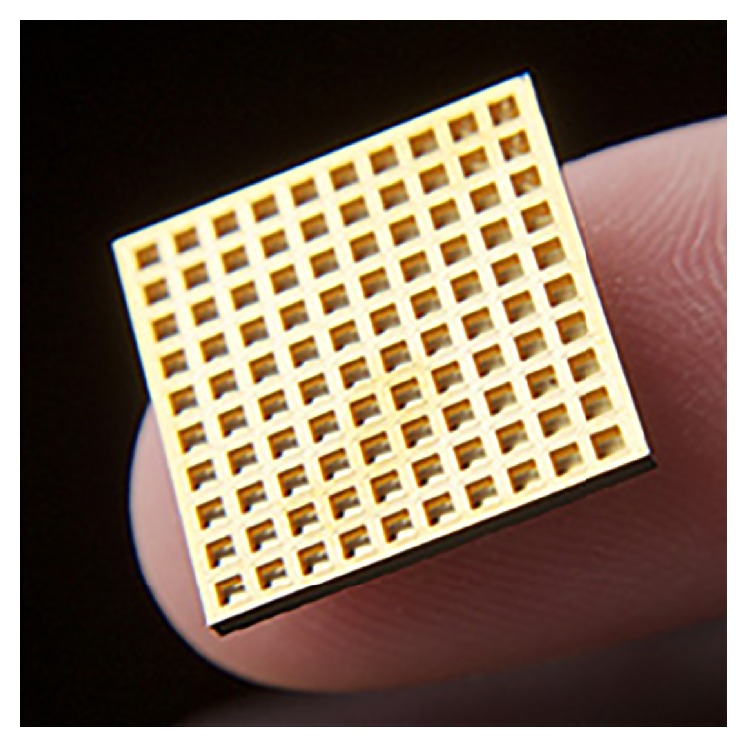
A microchip [24].

Implanted microchips enable on-demand drug release [5, 12, 13]. Solid silicon microchips consist of hundreds of reservoirs filled with up to 1 mL drugs in an aseptic solid, liquid, or gel filing [14, 15]. The multireservoir microchips are hermetically sealed to avoid degradation and subsequently covered by an anode membrane which can be ablated electrothermally to release the reservoir contents [16] (Figures 1 [Fig fig2]–3).

Understanding the components of microchips is best done in the context of how microchips are fabricated. Microchips are fabricated using the same well-developed technology as used for microelectronic integrated circuits and microelectromechanical systems (MEMS) [17], processes used to manufacture microdevices such as pressure sensors, accelerometers, flow sensors, inkjet printer heads, and micromirrors for projection [18]. To allow for accurate control of surface microarchitecture, microchips are created using repetitive thin-film deposition, photolithography, and etching (removing) [19].

Leveraging MEMS fabrication technology, the process begins with depositing an insulating or dielectric material on both sides of a substrate surface [20]. The substrate provides structural support to the device. Substrates have been made from ceramics, semiconductors, degradable polyethylene glycol, and most commonly silicon [21, 22]. Then using photolithography the insulating material is photomasked to a light-sensitive chemical resist onto the substrate, to pattern the desired geometric shape, serving as an etching mask. Various etching processes are used to generate the desired reservoir topography on one side of the insulator and substrate. On the yet-interrupted surface, an anode is created by laying electrode over the reservoir opening. The insulating material functions as the cathode. At the base of the to-be reservoir, the insulating material is removed and the reservoirs are filled with the drug solution of choice. Reservoir filling can be accomplished using injection/inkjet printing or spin coating methods. Wafer bonding—a method of hermetically encapsulating MEMS—is then used to cover and seal the reservoir [23].

### 2.2. Controlled Release

Microreservoir release is achieved by applying a voltage between the thin, metallic (e.g., copper or gold) anode membrane and a cathode to electrochemically dissolve the reservoir cover. This electrical potential can be activated wirelessly, external to the body, or secondary to metabolic changes in the host. The control circuitry can be integrated into the microchips. This circuitry includes a timer, demultiplexer, microprocessor, and input source (e.g., biosensor) [11]. Such controlled drug delivery can release drugs over months, on a preset or as needed schedule [24, 25].

## 3. Current Applications: Recent Studies and Patents

A survey of recent microchip developments, notable patents, and clinically relevant applications can inform about the position of microchips in medicine today, as well as motivating areas of further study. In 1998, the US Patent “Microchip Drug Delivery Devices” was awarded to Santini Jr. et al., which first outlined the parameters of a multireservoir microchip system with an active release system [26]. In 1999, Santini Jr. et al. debuted the first electrochemically activated drug delivery microchip [12]. In their device, release from individually dosed reservoirs is activated by applying an electric potential between the cathode and the anode—a thin gold membrane covering the specific reservoir to be deployed [12]. An extensive number of further advancements, notably including (but not limited to) refined fabrication methods [27], microchip flexibility for ophthalmic use and improved versatility [22], methods of operation [28], and details of wireless data and power transfer [29], have since been discovered and patented by the group.

**Figure 4:**
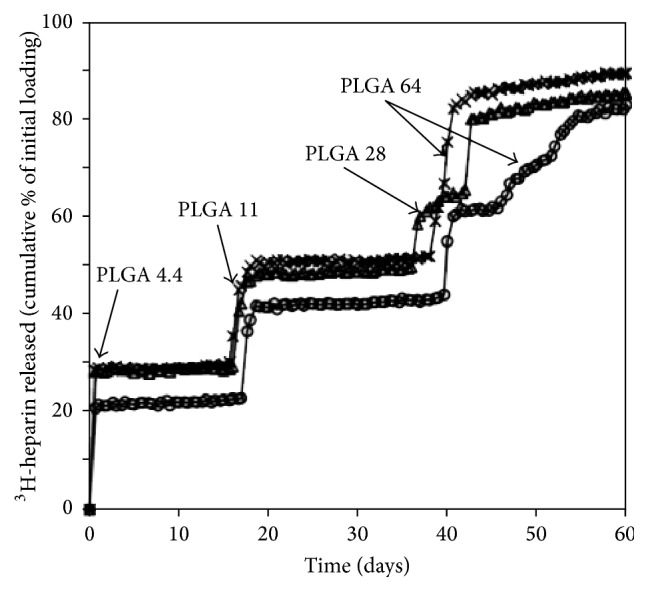
Each symbol represents the testing of a single device. Each device was loaded with four drug reservoirs. For all drugs utilized, “the release times of the chemicals from the reservoirs increased as the molecular mass of the reservoir membrane polymers was increased” [5].

Building on these studies, initial* in vitro* release studies determined whether microchip technology could achieve controlled release of a chosen therapeutic agent, with regular pulses of drug expulsion into the experimental system (Figure 4) [5]. Various molecular masses of PLGA copolymer (PLGA 4.4, 11, 28, or 64) were chosen as the reservoir membrane material of choice, with a 50 : 50 ratio of lactic acid and glycolic acid [5]. As seen with ^3^H-heparin release below, a consistent stepwise release of the drug was observed—correlating with each of the microchip reservoir membranes degrading and opening [5]. Similar results were achieved with ^14^C-dextran, ^125^I-HGH, and a combination of dextran and heparin. These findings provided evidence that controllable and pulsatile drug release from microchips was achievable* in vitro*, catalyzing* in vivo* experimental models. A 2007 study investigated the canine pharmacokinetic profiles of leuprolide—a polypeptide therapeutic indicated for prostate cancer and endometriosis treatment—when delivered* in vivo* via microchip reservoirs compared to subcutaneous injection [13]. The authors concluded that the pharmacokinetics of the two delivery methods were indeed comparable, yet the microchip method offered greater control over serum drug concentration [13]. Interestingly, the fibrous capsule that formed around the implant was found to not significantly affect the pharmacokinetic parameters of the drug—a notable consideration for human* in vivo* use, where bioadhesion to foreign material (conducive to equipment malfunction, altered performance, infection, etc.) is well documented.

### 3.1. Brain Cancer

**Figure 5:**
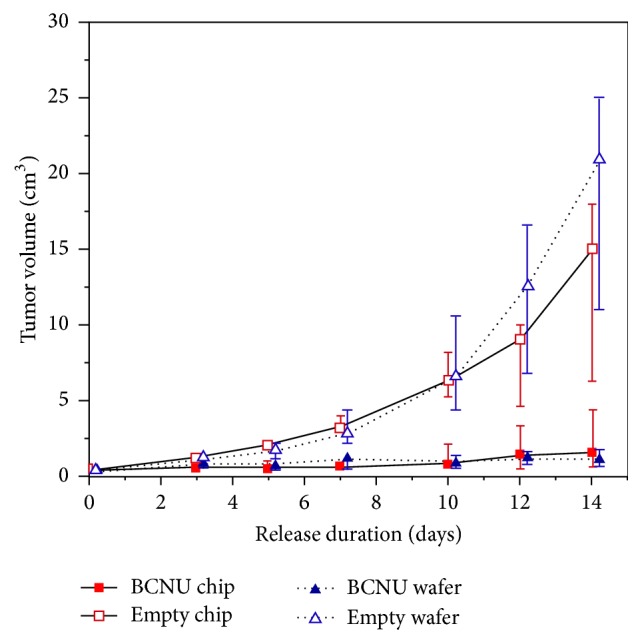
In comparison to the standard of care (inserted polymeric containing wafers containing the chemotherapeutic BCNU) microchip-mediated BCNU release achieved comparable levels of tumor volume suppression in the flank of rats [30].

The first investigation of polymer microchip* in vivo* efficacy for brain cancer therapy provided evidence that microchips, paired with the correct application and therapeutic agents, could be clinically implemented. Varying doses of 1,3-bis(2-chloroethyl)-1-nitrosourea (BCNU), a brain cancer chemotherapeutic, were loaded onto microchips and subsequently implanted into the flank of rats, where gliosarcomas had been introduced (Figure 5) [30]. The microchip-mediated BCNU effect on tumor size was compared to the standard of care, BCNU delivery from homogenous polymer wafers [30]. By measuring the concurrent tumor size with treatment, the authors concluded that the microchip BCNU release matched the efficacy of the polymer wafer in a dose-dependent manner [30]. As seen in Figure 5, the BCNU chip achieved comparable suppression of tumor volume. Applications within chronic conditions such as brain cancer, in which continuous and controllable local drug delivery to a difficult-to-access anatomical location is desired, indicate promising emerging fields for microchip technology.

### 3.2. Osteopenia and Osteoporosis

**Figure 6:**
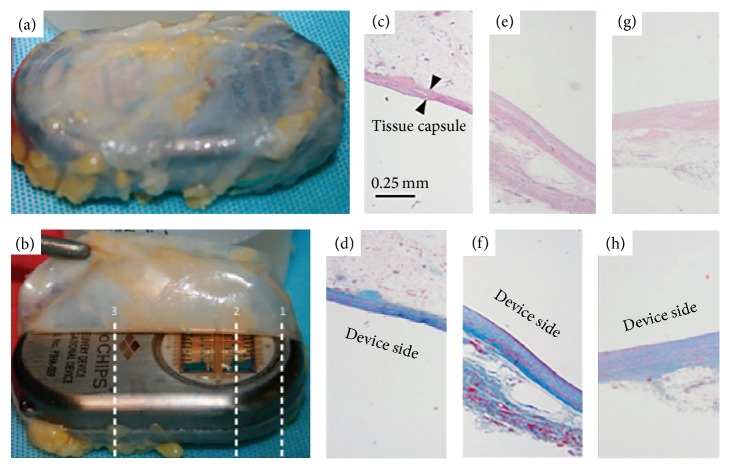
Biocompatibility of the implant was demonstrated, with normal immune responses and wound healing markers observed in six of seven implants. In addition, the release kinetics of the drug (along with its efficacy) were not impaired despite tissue capsule formation around implant [25].

In 2012, Farra et al. investigated the human* in vivo* pharmacokinetics of human parathyroid hormone, hPTH(1-34), released from microchip devices in eight female patients with osteopenia or osteoporosis (Figure 6) [25]. Release from the devices was activated 8 weeks after implantation, to allow for formation of a tissue capsule [25]. The pharmacokinetic profiles of the parathyroid hormone released were found to be reproducible day-to-day by device and bioequivalent in comparison to injections of FORSTEO, the on-the-market hPTH(1-34) treatment [25]. Biomarkers of skeletal response and bone formation closely paralleled PK findings, and total biocompatibility, safety, and patient satisfaction were also documented [25].

### 3.3. Abdominal Implantation

A concern of implementing microchips for drug release is the development of a tissue capsule around the microchip device, which could alter the release kinetics of the bioactive agent and decrease efficacy. Based on serum samples, the investigators demonstrated that release kinetics were comparable to injections. Taking into consideration the potential deleterious effects or immune responses from the implant itself, the investigators subjected the microchip (and tissue capsule) to histology testing upon removal from the abdominal cavity (photo of microchip and tissue capsule is shown in Figure 6). Six of the seven capsules demonstrated normal wound healing responses, with healthy levels of inflammatory cells. The seventh capsule histology sample indicated an elevated level of macrophages but was still within normal limits [25]. These findings helped to further assuage concerns regarding the viability of microchip usage in humans.

In summary, the largest drawback to the study was found to be overall equipment functionality. One of the eight implanted devices failed to release any drug and was thus excluded from analysis [25]. Despite this malfunction, this landmark study demonstrated the convenient and efficacious application of microchips in medicine, as an alternative to treatment with multiple regular injections.

## 4. Future Applications

The widespread application of microchip technology has the potential to be transformative to the modern healthcare system. Therapeutic processes will be changed, billions of dollars worth of unnecessary expenses will be avoided, and the quality of life of patient populations will increase.

While human studies involving microchips have been limited to treating a few specific diseases, advancements will allow expansion of this technology into a larger range of therapeutic areas. Drugs with dose delivery systems which would otherwise be considered difficult or undesirable could take place in passive manners. Treatments for diseases such as diabetes and hypertension where dose titrations are necessary could be revolutionized to create automated therapy regimens that are safer and more efficacious. When used in conjunction with implants, this controlled-release technology will decrease the likelihood of foreign body responses and rejection, therefore lowering the probability of inflammation and pain, allowing the body to heal faster after surgery. Applications of microchips could be extended to create artificial glands. Regulations of hormones in the body associated with dysfunctional glands could aid in both controlling current disease states and preventing the onset of other hormone prompted disorders.

Microchip delivery systems will aid in the treatments for diseases that classically include a lower rate of compliance (mental disorders, some cancer therapeutics, long-term antibiotics, etc.) or potential for abuse. An expansion in patient compliance will save billions in healthcare expenses every year through the reductions in hospital stays, doctor visits, and failures to follow prescriptions. Drug abuse could be better regulated for patients who receive schedules II and III classified treatments. Patients with addiction prior to implantation could be weaned off of their medication until they receive the intended set of benefits.

With advances in microchip technology itself, as well as trials demonstrating pulsatile release, stable drug pharmacokinetics, and utility and efficacy in treating disease states, microchip applicability is on the rise. Further research is required to establish clinical settings in which a drug (or health condition) requires local release, pulsatile control, and/or decreased compliance burden. Since the anode membrane is ablated electrothermally, the fate of the degraded byproducts on drug release, compatibility, and toxicity requires additional investigation* in vivo*.

Diabetes serves as the primary cause of death for 69,071 Americans every year, making it one of the top ten killers in the US. While still in development stages, microchip technology will have a large impact on the diabetes treatment landscape, saving the lives of hundreds of thousands of people. Current diabetes treatments are largely limited by delivery methods. Oral therapies offer a low bioavailability and relatively delayed impact. While liquid insulin (in the form of pumps or syringes) has a high bioavailability and fast entrance into the systemic circulation, patients are often deterred from considering it as a therapy option because of the need for self-injection. Furthermore, error in treatment occurs frequently with both methods of insulin therapy when patients give themselves the wrong dosage or forget to test their blood.

Construction of a high-level market model to forecast the future sales for a company that utilizes microchip technology begins with a measure of the size of the patient population and current statistics regarding treatment options. From these projections, estimates of conversion rates, pricing, and raw peak sales are determined (Table 1). Raw peak sales were calculated for the year 2035, which would be reasonable if preclinical trials were currently underway. This estimate was used for the sake of modeling potential sales since no date is known for the start of research into its application in the diabetes field. Though the technology already has evidence supporting its efficacy, its modeled early stage in development and the large number of competitors in the insulin market yield both a low probability of success and a low market share. With these numbers handicapping the sales projections, Company X would still be near $1 billion in peak sales, giving the application of microchip technology in the field of diabetes a blockbuster status.

While the financial modeling in Table 1 is used in the context of one specific industry, microchip technology can be utilized in a number of different facets of the modern healthcare system. As a disruptive technology, microchips would produce analyses that would yield similar degrees of impacts on their respective industries.

## Figures and Tables

**Table 1:** Market model, insulin for types 1 and 2 diabetes. Diabetes statistics providing estimates for population sizes were collected from the American diabetes association [31].

Microchips in insulin delivery for types 1 & 2 diabetes for Company X	
US diabetes type 1 prevalence (millions)	1.75
US diabetes type 2 prevalence (millions)	21
*Total (millions)*	*22.75*
Annual increase in diabetes population (millions)	1.4
Annual decrease in diabetes population (millions) (mortality rate *∗* diabetes prevalence)	0.2418
*Annual increase in prevalence (millions)*	*1.1582*
Delivery methods	
Insulin only	14%
Oral only	56.90%
Insulin & oral	14.40%
Other	14.70%
*Total on insulin*	*28.40%*
^*∗*^ Insulin only refers to both pump and syringe treatments	
Average conversion rate to microchip therapy	
Insulin only	25.00%
Oral only	10.00%
Insulin & oral	17.50%
Other	2.50%
*Percentage of population on microchip*	*12.0775%*
Pricing of microchip therapy^*∗*^ (2015 dollars)	
*Annual per patient cost*	$*10,000*
^*∗*^Based upon top line pricing of insulin pump treatments & insertion procedures	
Raw peak sales^*∗*^	
*Year 2035 (millions)*	$*55,452.63*
^*∗*^Accounting for increase in patient population size	
Probability of success	15%
Share of market	10%
*Company X peak sales (millions)*	$*831.79*

^*∗*^Preceding assumptions.
